# Fabrication of Electrochemical Biosensor Based on Titanium Dioxide Nanotubes and Silver Nanoparticles for Heat Shock Protein 70 Detection

**DOI:** 10.3390/ma14133767

**Published:** 2021-07-05

**Authors:** Marta Nycz, Katarzyna Arkusz, Dorota G. Pijanowska

**Affiliations:** 1 Department of Biomedical Engineering, Faculty of Mechanical Engineering, University of Zielona Gora, Prof. Z. Szafrana 4, 65-516 Zielona Gora, Poland; k.arkusz@ibem.uz.zgora.pl; 2 Nalecz Institute of Biocybernetics and Biomedical Engineering, Polish Academy of Sciences, Ks. Trojdena 4, 02-109 Warszawa, Poland; dpijanowska@ibib.waw.pl

**Keywords:** cyclic voltammetry, electrochemical impedance spectroscopy, electrodeposition, electroreduction, heat shock protein 70 (HSP70), silver nanoparticles (AgNPs), titanium dioxide (TiO_2_), titanium nanotubes (TNTs)

## Abstract

This paper presents the fabrication methodology of an electrochemical biosensor for the detection of heat shock protein 70 (HSP70) as a potential tumor marker with high diagnostic sensitivity. The sensor substrate was a composite based on titanium dioxide nanotubes (TNTs) and silver nanoparticles (AgNPs) produced directly on TNTs by electrodeposition, to which anti-HSP70 antibodies were attached by covalent functionalization. This manuscript contains a detailed description of the production, modification, and the complete characteristics of the material used as a biosensor platform. As-formed TNTs, annealed TNTs, and the final sensor platform—AgNPs/TNTs, were tested using scanning electron microscopy (SEM), X-ray photoelectron spectroscopy (XPS), and X-ray diffraction analysis (XRD). In addition, open circuit potential (OCP), electrochemical impedance spectroscopy (EIS), and cyclic voltammetry (CV) of these substrates were used to assess the influence of TNTs modification on their electrochemical characteristics. The EIS technique was used to monitor the functionalization steps of the AgNPs/TNTs electrode and the interaction between anti-HSP70 and HSP70. The produced composite was characterized by high purity, and electrical conductivity improved more than twice compared to unmodified TNTs. The linear detection range of HSP70 of the developed biosensor was in the concentration range from 0.1 to 100 ng/mL.

## 1. Introduction

The 2019 report on the global electrochemical sensor market published by Mordor Intelligence [1] estimated its value at USD 6.19 billion, and is expected to reach 11.83 billion by 2026, with an annual growth of 11.4% over the 2020–2025 forecast period. The sensors of that type owe their popularity to price competitiveness, high sensitivity, and simplicity of construction, including the ease of miniaturization. In addition, they can provide real-time information on the sample with or without minimal prior preparation. These features make the electrochemical sensors an excellent complement or alternative to chromatographic and spectrometric methods requiring a specialized laboratory, making them expensive, time-consuming, and interference-prone [2].

The greatest benefits of using this type of device are attributed to the manufacturing, chemical, and marine sectors in the monitoring of toxic and flammable gases; to the automotive sector in air quality or fuel-efficiency control; and to the medicine sector, driven by the need to implement non-standard diagnostic methods for effective identification or monitoring various diseases in real-time, especially for point-of-care applications. Currently, the development of electrochemical sensors is focused on the study of new materials and the use of the combinations of these materials (composites, hybrids) in order to improve specific properties, such as the development of new production methods (cheaper, faster, and non-requiring the use of many reagents and devices) and strategies to improve their selectivity and detection limits [1,3].

After discovering carbon nanotubes (CNTs) by Iijima [4], the combination of shape and interesting functionalities, which can be directly influenced by changing the geometry of the CNTs, has involved researchers in many fields of science. However, the biggest drawback of most carbon-based nanomaterials (like carbon nanoparticles and CNTs) is their toxicity. Therefore, there has been an unflagging interest in titanium dioxide nanotubes (TNTs) for the last 20 years [5,6,7]. Among all transition metal oxides, titanium dioxide (TiO_2_) shows a wide range of functional properties, such as chemical and thermal stability, corrosion resistance, biocompatibility, and good electrical conductivity [8]. Nanostructured TiO_2_ has additional advantages, and the most important of them are large surface area, interesting electronic properties, hydrophilicity, ease, and low cost of production [9,10]. Their production ensures the greatest control of nanotube dimensions by electrochemical anodizing of titanium in an electrolyte with fluoride ions [6]. This method results in an ordered arrangement of nanotubes (self-organization) attached directly to the titanium foil, which ensures their electrical connection with the substrate [6,10].

As is well known, each material has its strengths and weaknesses. By combining two or more components, we can obtain composites with the properties that are a combination of the advantages of their components and compensate for the disadvantages of each of them. In the context of nanoTiO_2,_ its electrical conductivity is most often improved in sensor applications. Therefore, with the development of nanotechnology, in addition to improving conductivity through heat treatment, in recent years, it has become popular to modify them by decorating with transition metals (including very popular precious metals, i.e., gold and platinum), non-metals, and also nanostructures—especially semiconductor quantum dots or carbon nanomaterials [11,12,13,14]. However, the literature contains many studies on TiO_2_ nanotubes as substrates for various types of sensors [15], only a few reports related to their use in this area combined with AgNPs [16,17]. Silver has the highest electrical conductivity among all metals, so that AgNPs can facilitate electron transfer more efficiently than other nanoparticles [18]. Moreover, their advantages include low-cost and straightforward production and the efficient combination with proteins by reaction with a thiol group (-SH) [19,20]. These features make AgNPs a promising material to be used in the design of electrochemical sensors [20,21]. The main difficulty/challenge in producing these types of composites is to obtain structures with high chemical purity, as the most common methods of their production, i.e., the chemical reduction required the use of reagents in the form of reducers, stabilizers, and other auxiliary substances can contaminate them [22,23]. An additional obstacle is the multi-stage nature of this process and the stability of the AgNPs-TNTs connection. Therefore, it seems that electro-reduction methods can be an alternative to complex chemical methods, as they guarantee a fast, highly productive production of stable AgNPs directly on TNTs. Additionally, these techniques are easily controllable and do not require high temperatures [20,24].

The global cancer burden is expected to be 28.4 million cases by 2040, a 47% increase from 2020 [25]. Several dozen markers are known currently, but only a few of them are considered truly reliable, so the search for new ones is a continuous, ongoing process. Many of the most frequently determined cancer markers are characterized by low diagnostic sensitivity in the early stage of the disease, e.g., CA (carcinoma antigen) 15-3 breast cancer marker [26], CA19-9 pancreatic cancer marker [27], PSA (prostate-specific antigen) prostate cancer marker [28], and SCC-Ag (squamous cell carcinoma antigen) cervical cancer marker [29]. Recent research suggests that the heat shock proteins (HSP), also called molecular chaperones, whose expression increases in response to exposure to stressful conditions (such as, among others, higher temperature, toxins, heavy metals, and alcohol) may constitute a marker of cancer aggressiveness or may enable monitoring of oncologic patients under treatment. Overexpression of HSP is observed in the case of several dozen types of cancer [30,31,32,33,34,35], including hematopoietic tumors, i.e., leukemia, multiple myeloma, breast cancer, cervical cancer, bladder cancer, renal cell carcinoma, liver cancer, lung cancer, oesophageal cancer, colorectal cancer, brain, and neck tumor including glioblastoma, pancreatic cancer, and prostate cancer. The authors [28,36,37] indicate the need for the simultaneous use of HSP70 and PSA as diagnostic and prognostic markers in the early stage of prostate cancer because, at the beginning of cancer disease, the level of PSA often does not change. At the same time, HSP70 shows high diagnostic sensitivity, even in the early stages of prostate cancer. Furthermore, many studies have indicated the correlation between these protein concentrations and the tumor malignancy level or the metastatic tumor potential [30,31,37]. Despite these facts, to date, only eight biosensors have been developed to determine the HSP70 level (Table 1), most of which use the optical detection method (surface plasmon resonance, SPR). Three of these biosensors used the electrochemical detection method [38,39,40]: cyclic voltammetry (CV) and electrochemical impedance spectroscopy (EIS), reached high detection limits, but their construction was made of expensive materials, such as glassy carbon electrode, indium tin oxide (ITO), graphene oxide and nanogold.

As an alternative, in the current work, we present an electrochemical composite biosensor based on titanium dioxide nanotubes and silver nanoparticles, providing fast and cheap HSP70 detection based on antibody-antigen interaction. The hypothesis of this work is as follows: titanium dioxide nanotubes doped with silver nanoparticles allow qualitative and quantitative detection of HSP70 with the use of the electrochemical impedance spectroscopy method. Our previous research [46] proved that this composite can be successfully used in simple detection systems (based on adsorption through intermolecular interactions) without the need to functionalize their surface. However, a change in pH or ionic strength or the presence of additives in the tested sample may desorb adsorbed proteins, which can be overcome using covalent functionalization [47]. The anti-HSP70 antibodies were therefore covalently immobilized with 11-mercaptoundecanoic acid (MUA) and N-(3-dimethylaminopropyl)-N′-ethylcarbodiimide hydrochloride (EDC) and N-hydroxysuccinimide (NHS). This manuscript contains a detailed description of the biosensor platform’s production, modification, and complete material characteristics. The EIS technique was used to monitor the functionalization steps of the AgNPs/TNTs electrode and the interaction between biomolecules. It can be concluded that the produced electrode allowed the successful immobilization of the anti-HSP70 antibody on the platform and the detection of HSP70 in the concentration range from 0.1 to 100 ng/mL. To the best of our knowledge, it is the first paper presenting an electrochemical biosensor based on AgNPs/TNTs to detect the HSP70 marker.

## 2. Materials and Methods

Titanium (Ti) foil (purity 99.7%, thickness 0.25 mm) and platinum mesh (purity 99.9%) were purchased from Sigma–Aldrich (St. Louis, MO, USA). All solutions were prepared using high purity reagents: ethylene glycol 99.8%, ammonium fluoride (NH_4_F) ≥ 98.0%, 11-mercaptoundecanoic acid (MUA) 95%, N-(3-dimethylaminopropyl)-N′-ethylcarbodiimide hydrochloride (EDC) ≥ 98.0%, N-hydroxysuccinimide (NHS) 98%, ethanolamine ≥ 99.0%, ethanol 96%, bovine serum albumin (BSA) ≥ 98%, phosphate buffered saline (PBS) (0.01 M, pH 7.4) from Sigma–Aldrich, silver nitrate (AgNO_3_) 99.9% from Stanlab (Lublin, PL) and hydrochloric acid (HCl) 99.9% from POCH (Gliwice, Poland). Monoclonal anti-heat shock protein 70 antibody (anti-HSP70) and heat shock protein 70 (HSP70) were purchased from Abcam (Cambridge, UK).

### 2.1. Fabrication of TiO_2_ Nanotubes

TiO_2_ nanotubes were formed on a titanium foil surface with electrochemical anodization method using the two-electrode system. The working electrode was a Ti foil, and the counter electrode was platinum mesh. Anodizing was carried out in ethylene glycol (85% wt.) with ammonium fluoride (0.65% wt.) at 17 V for 62.5 min using an Autolab PGSTAT302N (Metrohm, Herisau, Switzerland). Then, TNTs as-formed samples were annealed in an argon atmosphere at 450 °C for two hours with heating and cooling rates of 6 °C·min^−^^1^ using an AMP furnace (AMP, Zielona Gora, Poland).

### 2.2. Electrodeposition of Silver Nanoparticles on TiO_2_ Nanotubes

The deposition of AgNPs was carried out in a 1 mM AgNO_3_ solution in the potential range from −1.25 to −0.7 V with a scan rate of 50 mV/s for 25 cycles using an Autolab PGSTAT302N (Metrohm, Herisau, Switzerland) in a three-electrode system, in which the working electrode was TNTs on a titanium foil, the reference electrode was silver chloride electrode (E_Ag/AgCl (3 M KCl)_ = 0.222 V vs. SHE) by Metrohm, and the auxiliary electrode was platinum mesh. Next, the AgNPs/TNTs electrodes were carefully rinsed with distilled water and dried under a nitrogen stream.

The optimization of the nanoparticle deposition process aimed at obtaining a composite with the highest conductivity, as was presented in the authors’ earlier work [48]. In addition, the electrochemical stability [49] and antibacterial activity [50] of the composites were also assessed.

### 2.3. Covalent Immobilization of Anti-HSP70 on AgNPs Modified TNTs Electrodes

**Figure 1:**
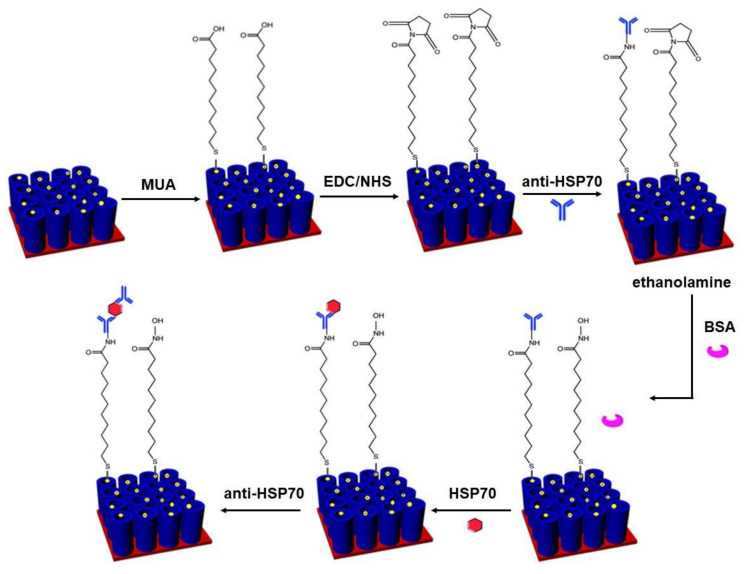
Schematic procedure of covalent functionalization of the AgNPs/TNTs biosensor for the determination of HSP70.

The AgNPs/TNTs was incubated overnight in 1 mM ethanolic solution of MUA. The sample was then rinsed with ethanol and distilled water and activated in a solution containing 0.4 M EDC and 0.1 M NHS (1:1) for one hour. Next, the electrode was washed with water, and 5 µL of 1 µg/mL anti-HSP70 solution in PBS was pipetted onto the surface. The immobilization of antibodies lasted one hour. One-hour surface inactivation was carried out with a 1M solution of ethanolamine in 1M HCl (pH = 8.5). The effectiveness of inactivation was verified by dropping and incubating the BSA solution on the AgNPs/TNTs surface. The detection of HSP70 was performed by placing different concentrations of HSP70 in the range from 0.1 to 100 ng/mL on the biosensor surface for 1 h. Finally, anti-HSP70 was re-pipetted onto the surface to confirm the antibody-antigen complex formation similar to the sandwich test. After each protein immobilization step, the electrode was rinsed with a PBS solution to remove physically adsorbed elements. The scheme of the biosensor functionalization is presented in Figure 1.

The selectivity of the AgNPs/TNTs platform was proven in our earlier work [46], in which this nanocomposite was used to detect bovine serum albumin in the presence of interleukin-6 (IL-6). After immobilization of the anti-BSA and then the BSA antigen, significant changes in impedance parameters were observed, while after IL-6 deposition, these changes were smaller than the standard deviation. Hence, we can conclude that there was no non-specific binding of IL-6. Therefore, based on these results, we assumed that AgNPs/TNTs will also be highly selective for HSP70.

### 2.4. Electrochemical, Chemical, and Morphological Characterization

TNTs electrodes before and after annealing and after modification with silver nanoparticles were characterized by measuring the open circuit potential (OCP) for 30 min, electrochemical impedance spectroscopy in the frequency range from 10^5^ to 0.1 Hz with a signal amplitude of 10 mV, and cyclic voltammetry in the potential range from −1 V to 1 V with a scan rate of 50 mV/s. All experiments were performed in 0.01 M PBS solution using a three-electrode system described in Section 2.2. After each step of surface modification, the biosensor impedance response measurements were carried out using the same parameters as indicated above.

Observation of TNTs and AgNPs/TNTs morphology was carried out using a scanning electron microscope (SEM, JEOL JSM-7600F, Tokyo, Japan). X-ray photoelectron spectroscopy tests were carried out in a PHI Versa Probe II Scanning XPS system using an Al Kα radiation (1486.6 eV) at a vacuum of <3 × 10^−9^ bar. The *x*-axis in the XPS spectra was calibrated over the C1s carbon peak (C-C) at 284.8 eV. Spectral alignment was performed using the PHI MultiPak software and the background subtraction method using the Shirley function. X-ray diffraction analyses were performed with a Panalytical Empyrean diffractometer using Cu Kα radiation at 40 kV and 40 mA (1.540508 Å).

## 3. Results and Discussion

### 3.1. Electrodeposition of AgNPs on TNTs

**Figure 2:**
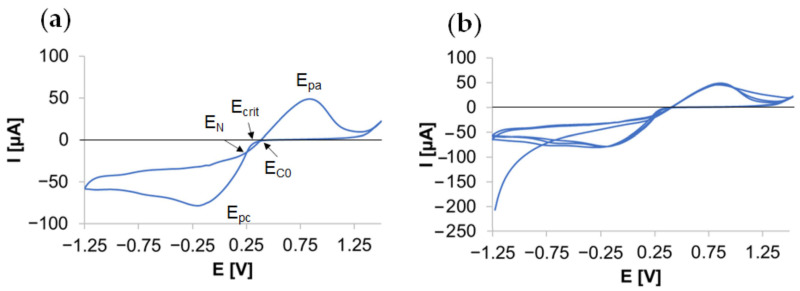
Cyclic voltammogram measured in 1 mM AgNO_3_ solution at a scan rate of 50 mV/s of a three-electrode system using annealed TNTs as the working electrode, silver chloride electrode (E_Ag/AgCl (3 M KCl)_ = 0.222 V vs. SHE) as the reference electrode, and Pt mesh as the auxiliary electrode (**a**) and three subsequent CV cycles (**b**).

Figure 2a shows the cyclic voltammogram recorded for the TNTs electrode in the AgNO_3_ solution. Its analysis is helpful in understanding the reactions involved in the AgNPs deposition process. The potential at which the recorded current decreases (0.3 V) is the critical nucleation potential (E_crit_), which means that deposition of silver cannot occur with more positive potentials [51]. The cathodic current at a potential of 0.25 V indicates the initiation of nucleation. The cathodic peak (E_pc_) at 0.2 V corresponds to the maximum reduction of silver, then the current decreases, which is related to the depletion of the amount of silver at the electrolyte/surface interface, and indicates a diffusion-controlled nucleation and growth mechanism. With the potential of −1 V, the slight increase in current is attributed to the hydrogen evolution reaction. After changing the scanning direction, two curve intersections are observed (E_N_ and E_C0_). The E_N_ potential (0.2 V) is the cross potential at which nucleation and growth occur at a measurable rate known as the nucleation overpotential [52,53], while the E_C0_ potential (0.35 V) is defined as the cross potential at which silver begins to reduce. The difference in the potential between E_N_ and E_C0_ results from a crystallographic mismatch between Ag and Ti. Hence, the presence of a hysteresis loop is an indicator of the formation of silver nuclei on TNTs. At more positive potentials than E_C0_, the silver begins to dissolve, and the current increases until the anodic peak (E_pa_) at 0.8 V, then it decreases, but the current intensity in the anodic half-wave potential does not reach 0 µA. The subsequent CV cycles (Figure 2b) do not completely overlap due to the remaining nucleation centers. This confirms that the silver particles did not dissolve completely when the scanning direction was reversed [52,54,55,56].

The studies by Bian et al. [57] on the electrodeposition of AgNPs on ITO showed that, with the increase of the deposition overpotential, the density of nanoparticles increased, and their size decreased. The highest density of the produced particles (which is related to the greater number of areas activated on the surface) and small dispersion of their size were obtained for high overpotential values ranging from −1.2 to −1.4 V. Moreover, the research by Plyasow et al. [58] on the deposition of nano platinum on glassy carbon and gold proved that when the deposition occurs in the range of potentials, at which adsorption and hydrogen release reactions also take place, these processes (on a competitive basis) inhibit the growth of platinum particles. Hence, in this study, it was decided to produce Ag nanoparticles in the potential range from −1.25 to −0.7 V.

### 3.2. Morphology of TNTs and AgNPs/TNTs Platforms

**Figure 3:**
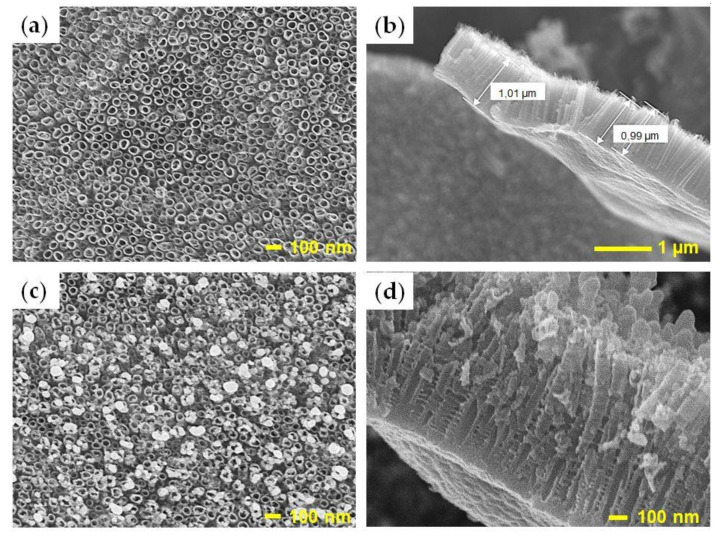
SEM top-view (**a**) and cross-sectional images of annealed TNTs (**b**), top-view (**c**) and cross-sectional images of AgNPs/TNTs (**d**).

Observations with a scanning electron microscope (Figure 3) showed that anodizing resulted in the formation of a layer of cylindrical titanium dioxide nanotubes with sidewall ripples (also called rings), which formed bridges between the adjacent nanotubes [59]. Thermal modification of TNTs did not damage their structure or change their dimensions, which is in line with the studies by Zhu et al. [60]. The outer (D_0_) and inner (D_1_) nanotube diameter were 50 ± 7 nm and 40 ± 3 nm, respectively, and the height of the formed oxide layer (L) was 1000 ± 68 nm. The specific surface area (A_s_), the total surface area of the nanotube (A_i_), and the number of nanotubes per square centimeter (n) were calculated from the following equations [61]:

(1) $$A_{s}={\mathrm{nA}}_{i}$$

(2) $$A_{i}=2\pi \left(D_{0}{}^{2}-{\;D}_{1}{}^{2}\right)+2\pi L\left(D_{0}+D_{1}\right)$$

(3) $$n=\frac{10^{14}}{{\left(3\right)}^{0.5}\times D_{0}{}^{2\;}}$$

The anodized surface area of the samples was 0.25 cm^2^, while the specific surface area of TiO_2_ nanotubes in this area was approx. 33 cm^2^; it follows that electrochemical oxidation increased A_s_ by approx. 132 times.

The AgNPs modified TNTs platform contained spherical and quasi-spherical particles ranging from 5 to 40 nm. The AgNPs electrochemical deposition process did not damage the ordered structure of TNTs and did not change the dimensions of the nanotubes. The nanoparticles were concentrated mainly on the surface of the nanotubular structure, mainly at the edges of TNTs, which is due to the higher electric current density in these regions. Therefore, their distribution was uniform and dense [46,62].

The mechanism of AgNPs formation and deposition onto TNTs consists of the silver nuclei formation, according to the Equations (4) and (5):

(4) $${\mathrm{AgNO}}_{3}\;\to {\;\mathrm{Ag}}^{+}+{\mathrm{NO}}_{3}^{-}$$

(5) $${\mathrm{Ag}}^{+}+{\;e\;}^{-}\to {\;\mathrm{Ag}}^{0}$$

Ag^+^ gains electrons near the substrate and undergoes reduction. As the process continues, the concentration of silver ions around the electrode decreases, resulting in a concentration gradient between the solution and the TNTs surface. Under the influence of the gradient force, numerous ions move towards the electrode, and consequently, more reduced Ag^0^ is formed on its surface [62]. Free atoms collide with each other and form nuclei with the diameter of several nanometers, becoming sites of nucleation and growth of silver nanoparticles by further reduction of cations. Self-organization of TNTs ensures a homogeneous environment for adatom deposition and the growth of Ag nuclei, whereas the upper edges of the nanotubes guarantee a multitude of nanoparticle nucleation sites. Interestingly, the studies of Yang et al. [63] showed that, under the same deposition conditions, and in the absence of TNTs, no nanoparticles were formed on the titanium foil, which indicates that nanotubes support their deposition.

In the initial stage of the electrodeposition, at the nucleation sites, silver builds up and form agglomerates. However, under the given current conditions, AgNPs are evenly distributed on TNTs after some time. As the process continues, re-aggregation of AgNPs may occur, and thus agglomeration may occur due to the saturation of the substrate [57]. A similar mechanism is described for the production of AgNPs on TNTs by chemical reduction [64].

Moreover, this process is similar to the formation of TiO_2_ nanotubes on titanium, which initially resembles a highly disordered porous structure. Then, the layer self-assembles as a result of equal current distribution between the pores and nanotubes are formed. However, increasing the anodizing time causes the upper boundaries of the nanotubes to dissolve and form “nanograss”.

Agglomeration of AgNPs can be explained by the difference in charge of AgNPs of differing sizes, described by Redmond and Brus [65] and Farkhondehfal et al. [66]. The larger Ag particles have a partial negative charge, and the smaller ones have a positive charge, therefore on the TNTs surface, the larger nanoparticle receives an electron from the adjacent smaller particle through the conductive substrate. At this point, the smaller nanoparticle becomes more positively charged and restores the electrical equilibrium by dissolving the Ag^+^. The result is an agglomeration of larger Ag nanoparticles and a dissolution of smaller ones (a kind of Ostwald ripening).

### 3.3. Chemical Analysis of TNTs and AgNPs/TNTs Platforms

**Figure 4:**
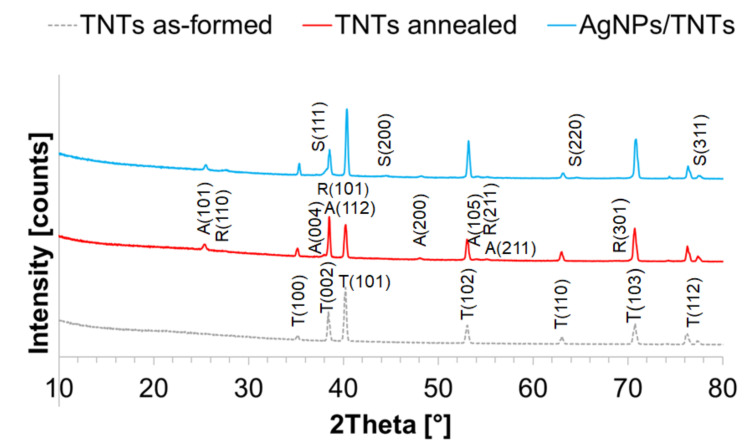
X-ray diffraction patterns of as-formed TNTs, annealed TNTs, and AgNPs/TNTs platform.

XRD diffractograms with the description of the peaks corresponding to the crystallographic planes for as-formed TNTs, annealed TNTs, and AgNPs/TNTs platforms are shown in Figure 4. XRD diffractograms of the TNTs as-formed sample showed the presence of peaks originating from the titanium substrate. Additionally, a slight background elevation at approx. 22° indicates the presence of amorphous TiO_2_ [67]. Amorphous materials scatter X-rays, which are manifested by broad humps on the diffraction patterns. In the case of the annealed platform, the presence of peaks originating from two crystal phases of TiO_2_, anatase and rutile, was also noted. The amount of anatase () in the structure of annealed TNTs was calculated from the Spurr and Myers equation [68]:

(6) $$A_{A}=\frac{1}{1+1.26\times \left(\frac{I_{R}}{I_{A}}\right)}$$

where: I_A_ is the intensity of the (101) peak of anatase, I_R_ is the intensity of the (110) peak of rutile. The anatase content in the annealed TNTs was predominant (53.9%), which is consistent with the results obtained by Huang et al. [69]. Notably, the results of the research by Liang et al. [64] indicated a weaker adhesion of silver nanoparticles to unannealed TiO_2_ nanotubes, and that the rutile structure can retard the formation of silver nanoparticles.

XRD diffractograms for AgNPs/TNTs, apart from the peaks derived from titanium substrate and TiO_2_ in anatase and rutile phase, showed the peaks for silver in the metallic form. The main silver diffraction peak (111—face-centered (fcc) cubic crystal lattice), which overlaps with both the peak for titanium (002) and anatase (112), reaches its maximum at approx. 38°. The main diffraction peak of silver (111), which coincides with titanium (002) and anatase (112) peaks, reaches their maximum at approx. 38° [70,71]. The highest intensity of this peak indicates that its (111) plane was the most preferred orientation because of its lowest free energy, since the Ag atoms had sufficient energy to move [64]. Importantly, only the characteristic Ag_2_O peak (at 32.85°) and no other additional peaks were observed in the diffraction patterns, which confirms the absence of oxidized forms of Ag [72] and the formation of undesirable by-products [70].

**Figure 5:**
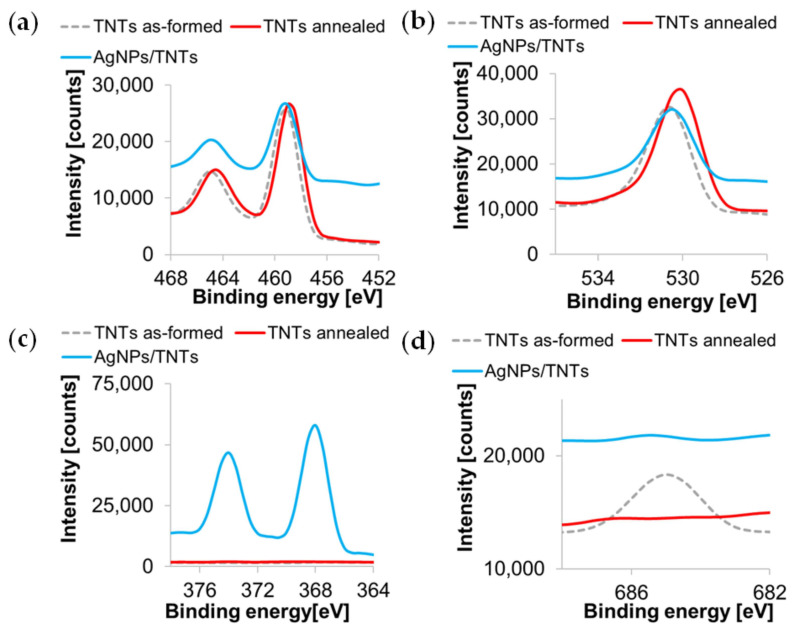
Ti 2p (**a**), O 1s (**b**), Ag 3d (**c**), and F 1s (**d**) XPS spectra of as-formed TNTs, annealed TNTs, and AgNPs/TNTs platform.

The summary of XPS spectra of titanium Ti 2p, oxygen O 1s, silver Ag 3d, and fluorine F 1s for as-formed TNTs, annealed TNTs, and AgNPs/TNTs are shown in Figure 5. Carbon, oxygen, and titanium were detected in all samples, as well as fluorine in as-formed TNTs and silver in the composite. The peak maximum for F 1s is observed at 684.2 eV. As it is present only in the as-formed structure, it can be stated that it exists in the form of F^-^ ions physically adsorbed to TNTs [73]. The thermal treatment resulted in the removal of fluoride ions from the structure. The Ti 2p peaks reached a maximum at 458.6 and 464.3 eV with a doublet separation equal to 5.7 eV, which indicates the presence of Ti^4+^ in TiO_2_ oxide. Moreover, the slight changes in the spectrum at 457.9 eV indicate the presence of Ti^3+^ in Ti_2_O_3_ [74]. The O 1s oxygen spectra for all samples are similar to each other, and their asymmetry indicates the presence of more than one type of bond. The peak at ~530 eV is attributed to oxygen in titanium oxides (O^2−^ in the crystal lattice) [75], while the distortion of the spectrum at higher binding energies corresponds to the OH^−^ groups or adsorbed water at point defects (oxygen vacancies), or oxygen in carbon contamination, which is recognized on the surface of most samples exposed to air [74,75]. The Ag 3d spectrum for the AgNPs/TNTs composite shows a 3d doublet structure (the d5/2—d3/2 separation is 6.0 eV), which indicates the metallic state of silver [70]. The Ag 3d5/2 peak is negatively shifted in relation to the standard binding energy (368.4 eV) due to the fact that spherical nanoparticles have a greater number of non-coordinated surface atoms, which reduces their binding energy [76,77]. Additionally, Figure 5a,b indicate a shift of the peak maxima to higher binding energy for the AgNPs/TNTs sample, compared to as-formed and annealed TNTs, due to the strong interaction between Ag and TiO_2,_ and indicates electron transfer between the structures [78,79,80]. According to the literature, this phenomenon results from the leveling of Fermi levels as a result of the contact of silver with TiO_2_ nanotubes [78,81]. Moreover, this can be interpreted as a partial reduction of Ti^4+^ on the surface, resulting from the formation of a metal-metal bond between Ag and Ti and a surface O-Ag bond. However, these shifts are not indicative of silver oxidation, which was also confirmed by XRD analysis [78,82].

The analysis of the chemical composition (Table 2), based on the XPS spectra, showed an increase of oxygen in the oxides and the loss of oxygen in the subsurface layer after thermal modification of TNTs, which is consistent with the study by Song et al. [82]. The ratio of oxygen in oxides to titanium is non-stoichiometric, indicating titanium oxides other than TiO_2_. The XPS analysis gives information on the composition of the surface layer to a depth of several nanometers. The reduction of titanium and oxygen in the composite (confirmed by the obvious reduction of the peaks intensity in Ti 2p and O 1s spectrum) indicates that TNTs has been modified by AgNPs mainly in the subsurface layer.

### 3.4. Electrochemical Analysis of TNTs and AgNPs/TNTs Platforms

**Figure 6:**
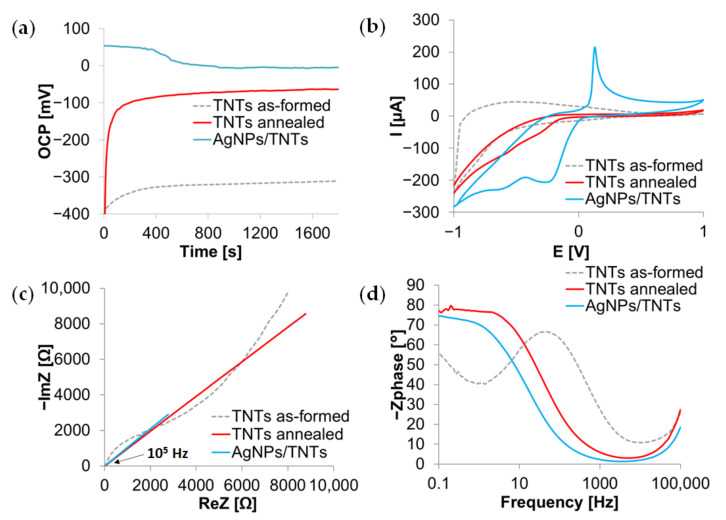
Open circuit potential curves (**a**), cyclic voltammograms (**b**), impedance spectra in Nyquist (**c**), and Bode (**d**) representation of as-formed TNTs, annealed TNTs, and AgNPs/TNTs platform, in PBS solution.

Open circuit potential measurements give information about the tendency of a material to corrode because its value indicates when corrosion may begin. Figure 6a demonstrates the stationary potential curves recorded during 30 min for as-formed TNTs, annealed TNTs, and AgNPs/TNTs composite. The OCP of all samples is relatively stabilized after about 500 s, proving the equilibrium between the dissolution of the oxide layer and the adsorption of ions on the tested surface. As-formed nanotubes are characterized by a negative value of the stationary potential, TNTs modifications increase OCP value and thus corrosion resistance [83]. The curve recorded for the AgNPs/TNTs shows mild oscillations, indicating that the processes taking place on the electrodes, i.e., the oxidation of silver on the surface, may result from the reaction of silver ions with chloride ions from PBS [49,84].

Cyclic voltammograms (Figure 6b) show significant current changes in the potential range from −1 V to −0.7 V, related to hydrogen evolution, and in the high potential range with surface oxidation—oxygen evolution. The waveforms recorded for the annealed and modified AgNPs samples additionally show the cathodic peak at approx. −0.5 V, which can be described as Ti^4+^ reduction to Ti^3+^ while a slight anodic peak at −0.2 V corresponds to the reverse reaction [85]. For AgNPs/TNTs, a clear oxidation peak at 0.2 V is associated with the formation of Ag^+^ ions, while the cleavage of the cathodic peak at −0.2 V is attributed to the Ag^+^ reduction [86]. The absence of additional peaks in the voltammograms indicates that no by-products are formed on the substrate. The separation between oxidation and reduction half waves is greater for the sample containing AgNPs, indicating higher conductivity of this electrode [64]. Small current values recorded at a more positive potential for TNTs samples are related to the fact that the nanotube walls were completely devoid of the free charge carrier, and the electron transport was controlled by the Schottky barrier formed at the metal/oxide interface [23,87]. The addition of silver accelerated the transport of electrons.

The Nyquist plots (Figure 6c) at the lowest frequencies show fragments not fully formed semicircles, characteristic for oxide layers [88], corresponding to the charge transfer resistance between the electrode and electrolyte, which decreases after heat treatment of the samples and modification of AgNPs. The linear characteristics obtained for annealed TNTs and AgNPs/TNTs indicate that these platforms allowed for faster electron transfer. The semicircle recorded for the as-formed TNTs platform in the high-frequency region indicates a high charge transfer resistance in the electrode material. The addition, AgNPs increased the conductivity of TNTs by about 50%. This is consistent with studies in which the addition of silver nanoparticles deposited on carbon nanotubes with reduced graphene oxide [89], graphite paste electrodes [90], or gold electrodes [91], which significantly accelerated the transfer of electrons. The improvement in conductivity after thermal treatment of TNTs results from the formation of oxygen vacancies and self-doping by the presence of Ti^3+^ ions, forming a donor level, thus lowering the TiO_2_ conductivity band and thus reducing the band gap [92]. Silver, as well as Ti^3+^, is an electron donor, hence its addition also reduces the size of the band gap [93].

The Bode plots in Figure 6d show the presence of two-time constants (maxima). The first-time constant occurs at the highest frequency and corresponds to the nanotube layer (or in the case of composite—nanotubes with AgNPs), while the second occurs at a frequency of approx. 100–500 Hz for as-formed TNTs and at approx. 10 Hz for the annealed TNTs, with AgNPs/TNTs platforms indicating a barrier layer [61]. This constant shift towards lower frequencies indicates a lower charge transfer resistance and thus a higher conductivity of modified TNTs. The thermal treatment increased the phase angle value in the low frequency area, related to the phase transformation of amorphous TiO_2_. The ideal capacitive behavior for low frequencies is equated to a phase angle close to 90°. In the high-frequency region, which characterizes the part of the electrode in contact with the electrolyte, changes in the phase angle are much smaller, which may additionally confirm that the annealing did not affect the morphology of the nanotubes. The modification of TNTs with silver nanoparticles reduced the phase angle value both in the high and low frequency regions, which is in line with the results of impedance tests on porous titanium with the addition of AgNPs obtained by Fadlallah et al. [94]. This proves that the nanoparticles deposited on the TNTs’ surface and inside their structure.

### 3.5. Functionalization of AgNPs/TNTs by Anti-HSP70 Antibody and Electrochemical Detection of HSP70 Antigen

For the functionalization of the AgNPs/TNTs surface, a commonly used covalent coupling procedure was selected in accordance with the scheme (Figure 1) and methodology described in the experimental part (Section 2.3). The first step of electrode modification was to cover the surface with the self-assembled monolayer (SAM) with -COOH ends by incubation in MUA solution and forming S-Ag bonds (preceded by breaking the S-H bond). The samples were then placed in the EDC/NHS mixture. EDC is an activator of carboxyl groups, while NHS increases the coupling efficiency. The activation of -COOH groups leads to the formation of an unstable intermediate compound, which undergoes hydrolysis in the presence of water. The addition of the NHS results in a more stable ester, which reacts with primary amino groups in the protein to form stable amide bonds. Therefore, the final product is the same as using only EDC. However, the NHS increases the lifetime (stability) of the intermediate compound (NHS esters have a half-life of 4–5 h at pH 7.1, an hour at pH 8, and only 10 min at pH 8.6). In the next step, anti-HSP70 antibodies were immobilized on the substrate [40]. The unreacted ester groups were blocked with ethanolamine [95] to prevent non-specific antigen binding. BSA was then applied to check that all free binding sites were blocked. After this stage, the antigens and again the HSP70 antibodies were immobilized on the surface. The re-addition of antibodies was to additionally confirm the formation of the antigen-antibody complex in the previous step.

**Figure 7:**
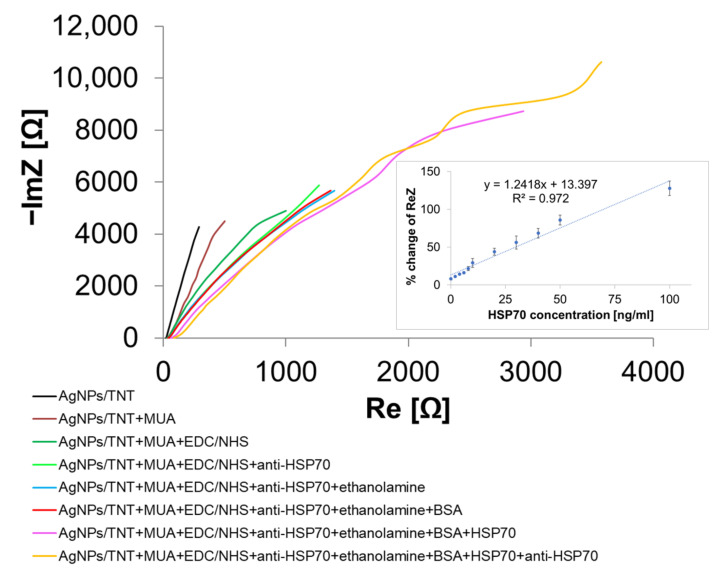
Nyquist plot showing immobilization steps (inset: HSP70 calibration curve obtained by the AgNPs/TNTs biosensor).

Figure 7a shows the Nyquist plot after the successive steps of the functionalization of the AgNPs/TNTs electrode and the immobilization of anti-HSP70 and HSP70 antigens. The impedance spectra in the Nyquist plot consisted of two parts: very small semicircles at the highest frequencies indicating an electron transfer controlled process, and parts of an incomplete larger semicircle corresponding to a diffusion-controlled process. The lowest impedance modulus value is observed for unmodified AgNPs/TNTs, proving the good conductivity of this substrate. After sample incubation in MUA, there was an increase in the real and imaginary parts of impedance, and therefore in the impedance modulus, which proves the formation of the SAM. The addition of EDC/NHS also blocked the kinetics of electron transfer. Similar changes were observed after the immobilization of anti-HSP70, as well as blocking the reactive sites of the substrate with ethanolamine. The lack of changes in impedance parameters after the immobilization of the BSA confirms the effective use of ethanolamine. Immobilization of the antigen resulted in rapidly increased resistance of the biosensor, which proves the antibody-antigen complex formation. Re-immobilization of HSP70 antibodies and the expected changes in impedance parameters confirmed the complex formation, which increased the thickness of the insulating biological layer. The demonstrated changes in impedance parameters after the functionalization are commonly observed in the literature and confirm that it was carried out correctly [38,39,40]. The HSP70 calibration curve (Figure 7) shows the relationship between the antigen concentration and the real part of impedance (ReZ). Changes in the ReZ parameter are given as percentages and represent the difference in the ReZ before the immobilization of the antigen on the substrate (after the immobilization of the antibody) and after anti-HSP70 dropping. The calibration curve shows the linear resistance response of the biosensor, with a correlation coefficient of 0.972. To calculate the limit of detection (LOD), the following equation [96] was used:

(7) $$\mathrm{LOD}=\frac{3.3\sigma }{s}$$

where: s is the slope of the calibration curve, σ is the standard deviation of response. The LOD was found as 0.48 ng/mL.

Repeatability tests were performed after each electrode modification step and consisted of EIS measurements using different electrodes. The relative standard deviations (RSD) of the ReZ impedance parameter for TNTs as-formed, TNTs annealed, and AgNPs/TNTs (PBS solution) were calculated as 14.5 ng/mL, 3.3 ng/mL, and 8.7 ng/mL, respectively. The low dispersion of the results is crucial when using platforms as a sensor. The heat treatment increased the reproducibility of the platform responses. The higher RSD value between different AgNPs/TNTs electrodes can be explained by the greater heterogeneity of the composite structure. The RSD of the biosensor response (at five different concentrations of HSP70: 0.1, 2, 30, 50, and 100 ng/mL) ranged from 1.8% to 9.22%, proving the reliability of the biosensor fabrication procedure.

It is worth noting that the AgNPs/TNTs nanocomposite has not been used to detect HSP70 so far. None of its components were used separately for this purpose. Most of the eight HSP70 sensors developed to date (Table 1) used an optical method based on the surface plasmon resonance, and their detection limit ranged from 0.1 to 1.29 µg/mL, while the norm in the serum of healthy people was up to 6.5 ng/mL. This means that their limit of detection exceeds the physiological level of HSP70 by at least two orders. In neoplastic diseases, HSP70 is overexpressed at the level of several dozen ng/mL. On the other hand, the three electrochemical sensors produced to-date were characterized by the determination of the level of femtograms. However, highly conductive and expensive materials have been used for their construction, i.e., ITO with AuNPs, or glassy carbon.

## 4. Conclusions

The aim of this study was to develop an electrochemical biosensor based on titanium dioxide nanotubes and silver nanoparticles to determine the concentration of a tumor marker, specifically heat shock protein HSP70, the overexpression of which is observed in the early stages of many neoplastic diseases. The produced composite sensor substrate was characterized by an even distribution of spherical and quasi-spherical silver nanoparticles on a self-organized nanotube structure. The annealed nanotubes had an anatase–rutile structure with a slight predominance of anatase. In addition, the XRD and XPS analysis confirmed the high purity of the produced nanostructures and the strong interaction between Ag and TiO_2,_ which resulted in improved electrical conductivity and corrosion resistance of the composite. Sensor detection was based on anti-HSP70—HSP70 interaction. The antibodies were covalently immobilized with MUA and EDC/NHS. The EIS technique was used to monitor the successive steps of the biosensor functionalization and the interaction between anti-HSP70 and HSP70. The created composite platform enabled the detection of HSP70 in the concentration range from 0.1 to 100 ng/mL. To the best of our knowledge, this is the first research paper presenting an electrochemical biosensor based on AgNPs/TNTs to detect the HSP70 marker.

## Figures and Tables

**Table 1:** HSP70 biosensors developed by 2021.

Detection Method	Electrode	Detection Limit	Ref.
Optical	SPR biosensor based on Au film	0.1 µg/mL	[41]
	SPR biosensor based on Au film with magnetic microbeads	0.3 µg/mL	[42]
	SPR biosensor based on Au film with titania sol-gel matrix and magnetic beads	0.1 µg/mL	[43]
	SPR biosensor based on glass with AuNPs	0.1 µg/mL	[44]
	Porous silicon-based biosensor	1290 ng/mL	[45]
Electrochemical	ITO coated PET biosensor with AuNPs	0.0618 fg/mL	[38]
	Graphene oxide modified glassy carbon biosensor	12 fg/mL	[39]
	Fullerene C60 modified glassy carbon biosensor	0.273 pg/mL	[40]

**Table 2:** Chemical composition (atomic%) of as-formed TNTs, annealed TNTs, and AgNPs/TNTs platform calculated from the XPS spectra.

	C_total_	F	O_in oxides_	O_non lattice/OH/H_2_O_	Ti	Ag^0^ (NPs)
TNTs as-formed	21.9	7.0	39.2	10.6	21.3	-
TNTs annealed	16.8	-	47.1	8.8	21.7	-
AgNPs/TNTs	21.8	-	32.4	7.0	16.2	22.6

## Data Availability

Not applicable.
